# Childhood Obesity and Overweight in Ghana: A Systematic Review and Meta-Analysis

**DOI:** 10.1155/2020/1907416

**Published:** 2020-04-08

**Authors:** Prince Kwaku Akowuah, Emmanuel Kobia-Acquah

**Affiliations:** Department of Optometry and Visual Science, Kwame Nkrumah University of Science and Technology, Private Mail Bag, University Post Office, Kumasi, Ghana

## Abstract

The increasing prevalence of childhood obesity and overweight is considered a public health issue in both developed and developing countries. This systematic review and meta-analysis estimates the prevalence of childhood obesity and overweight in Ghana. A multiple database search was conducted for articles published between January 1, 2001, and October 31, 2019, reporting the prevalence of childhood obesity and overweight in Ghana. Databases searched include PubMed, Google Scholar, Scopus, Cochrane Library, World Health Organization (WHO) Library Information System, and Africa Journals Online. Data were pooled from the articles to calculate an overall estimate of childhood obesity and overweight using a random-effects model after variance stabilization with Freeman–Tukey double arcsine transformation. This review adhered to the Preferred Reporting Items for Systematic Reviews and Meta-Analyses guidelines. Sixteen studies with a combined sample size of 29,160 were included in the review. Analysis indicates that approximately 19% of children in Ghana either have obesity or are overweight. The prevalence of childhood obesity and overweight was 8.6% (95% CI: 4.8%–13.4%) and 10.7% (95% CI: 5.9%–16.6%), respectively. Although not significant, higher obesity (4.6% vs. 2.6%) and overweight (11.0% vs. 7.2%) prevalence were estimated for females than for males. There was a significantly higher obesity prevalence estimate (17.4% vs. 8.9%) in rural settings than in urban settings (*p*=0.0255). The high prevalence of childhood obesity and overweight estimated in this review is of worrying concern. It is a significant public health problem that has implications on the health of present and future generations in Ghana and as such calls for proactive measures to be put in place. Also, the driving forces behind the increasing prevalence of childhood obesity in Ghana need to be investigated.

## 1. Introduction

Once considered a problem of Western countries, obesity has become a public health problem in low- and middle-income countries too [1–3]. Childhood obesity is of particular concern because it is associated with early onset of risk of diseases such as cardiovascular diseases and diabetes and higher odds of obesity in adulthood [4–7]. Childhood obesity has reached global epidemic levels and is a public health issue in both developing and developed countries. In 2016, approximately 41 million children under age 5 years were overweight or had obesity globally, about 25% of them in Africa alone [8]. The global prevalence of childhood obesity has been on the rise over the past decades, increasing from 4.2% in 1990 to 6.7% in 2010 and is expected to rise to 9.1% in 2020 [9].

Urbanization and economic development have influenced the increasing trend of adult and childhood obesity [10]. Urbanization has resulted in changes in dietary patterns in both developed and developing countries. Dietary patterns have shifted from traditional nutritious foods, which tend to be high in complex carbohydrate and vegetables, to foods containing high fat and calories [10–12]. In addition to changes in the type of food eaten, there have been changes in the timing of eating as more individuals are eating late into the night mainly due to night/shift work and increasing night lifestyle activities. Urbanization and economic developments have also led to increase in sedentary lifestyles. There has been a decrease in physical activities in both developed and developing countries [13]. Advancement in technology has led to relative ease of doing things and moving around; therefore, there is a considerable decrease in opportunities for physical activities [14, 15]. Children spend more time indoors than they do outdoors, playing video games, and partaking in other indoor activities [16–18]. The energy imbalance resulting from the increased high caloric food intake and decreased physical activities is the driving force behind the increase in obesity and overweight over the past decades [19].

The public health significance of obesity and overweight in Ghana is well recognized. Obesity and overweight in Ghana are estimated to have increased over the past decades [20]. The Ghana Demographic and Health Survey reported the increasing prevalence of childhood obesity in Ghana. Prevalence of childhood obesity in children under 5 years was estimated to have increased from less than 1% in 1988 to 5% in 2008 [21]. A 7% prevalence of obesity among children 13–15 years in Ghana was reported by the 2007 Global School-based Student Health Survey [22]. Over the past decades, different studies have reported varying prevalence values for childhood obesity and overweight in Ghana, in the range of 0.7%–47.06% for obesity and 0.8%–33.66% for overweight [23–26]. The varying prevalence of childhood obesity and overweight from the different studies is most likely due to the different geographical regions in which the different studies were conducted, as different regions of the country are known to have different dietary patterns and lifestyle [27–29]. Other possible reasons for the variations among these studies are difference in obesity and overweight diagnostic criteria used and different sampling periods. Despite all these reports and studies estimating the increasing prevalence of childhood obesity and overweight in Ghana, there is no current nationally representative dataset of childhood obesity in Ghana. Although a recent meta-analysis reported obesity and overweight in Ghana [30], this included only studies in Ghanaian adults. There remains no thorough systematic review and meta-analysis reporting the prevalence of childhood obesity and overweight in Ghana. An up-to-date and accurate documentation of prevalence of obesity and overweight in Ghana is needed to inform policy making as well as design and implementation of public health intervention programs. The objective of this review is to summarize and estimate the prevalence of obesity and overweight among children in Ghana using meta-analysis of the current available literature.

## 2. Methods

This review followed the recommendations in the Preferred Reporting Items for Systematic Reviews and Meta-Analyses (PRISMA) statement.

### 2.1. Literature Search Strategy

A systematic online search of primary literature on childhood obesity and overweight in Ghana was conducted. The databases searched were PubMed, Google Scholar, Scopus, Cochrane Library, World Health Organization (WHO) Library Information System (WHOLIS), and Africa Journals Online (AJOL). Different variations of search text were used in the literature search, each being an appropriate combination of any of the following words: “childhood OR pediatric OR juvenile OR infantile,” “prevalence” AND “obesity OR overweight OR adiposity OR malnutrition” AND “Ghana OR Ghanaian or Africa.” All literature searches were performed between 11/01/2019 and 11/15/2019. A secondary form of literature search was performed by reviewing all the references of the eligible articles that were obtained from the primary literature, for any relevant publication that might have been missed on the primary literature search.

### 2.2. Inclusion and Exclusion Criteria

Primary research articles published between January 2001 and October 2019 were included. This was to select articles reporting childhood obesity and overweight in Ghana within the 21^st^ century. Only studies involving participants 19 years and below were included. This was because for this review, we chose to define childhood using the WHO definition. Studies were included only if they reported the prevalence of childhood obesity and overweight separately and used body mass index (BMI) as a means of reporting the prevalence values. If a study does not report prevalence values for obesity or overweight but provides information with which obesity or overweight prevalence could be calculated, it was included. For data pooling, only studies that reported separate prevalence figures for childhood obesity and childhood overweight were included. Finally, only studies with full text of the publication were included.

### 2.3. Study Screening and Appraisal

For initial screening, studies were selected based on the set inclusion and exclusion criteria using their titles and abstracts. Full-text articles of studies that passed the initial screening were further screened to ensure all inclusion criteria were met. Information extracted from selected studies included authors' names, year of publication, sampling period, study location, sample size, study design, age range of participants, obesity and overweight diagnostic criteria used, and the prevalence of overweight and obesity. A 10-item checklist produced from the Downs and Black checklist [31], and the Strengthening the Reporting of Observational Studies in Epidemiology (STROBE) statement [32] was used to rate and assess the quality of all the full-text articles included in the review.

### 2.4. Data Analysis

OpenMeta (analyst), an open-source software for meta-analysis [33], and Comprehensive Meta-Analysis software were used for the statistical analysis. A 95% confidence interval was used in assessing the individual study proportion and pooled effects. The Freeman–Tukey double arcsine transformation was used before pooling to minimize the effects of studies with extremely high or low prevalence estimates on the overall pooled estimates [34]. Assessment of heterogeneity between studies was conducted using Cochran's heterogeneity statistics (Q) and degree of inconsistency (*I*^2^). The *I*^2^ statistic provides an estimate of the percentages of heterogeneity across studies that is truly due to differences between studies but not chance. *I*^2^ > 60% was regarded as meaningful heterogeneity. In cases of meaningful heterogeneity, analyses of pooled effects were performed with the random-effects model [35]. Meta-regression analysis was conducted to explore the association between factors such as gender, study setting, study location, and prevalence of childhood obesity and overweight. The robustness of the pooled effects and potential outliers were assessed with the leave-one-out sensitivity analysis. The leave-one-out sensitivity analysis evaluates the contribution of each study to the total pooled estimate [36]. The funnel plot was used to evaluate the presence of publication bias and confirmed by Egger's test. For all statistical analysis, a *p* value <0.05 was considered statistically significant.

## 3. Results

**Figure 1:**
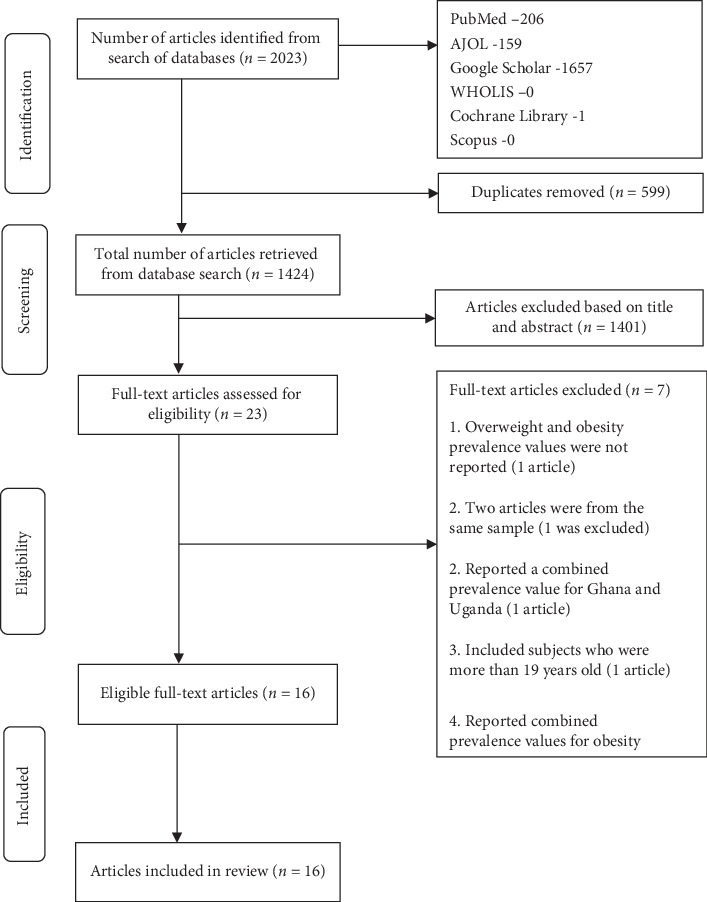
PRISMA flowchart of steps in identifying studies.

Two thousand and twenty-three (2023) articles were identified through search of the database. Five hundred and ninety-nine (599) were identified as duplicates and excluded based on their titles. Through screening against the preset criteria, 1401 articles were excluded based on their titles and abstracts, leaving 23 full-text articles to assess for eligibility; articles excluded at this stage were either from studies not conducted in Ghana, studies conducted in Ghana but in adults, or studies conducted in Ghana but the focus was not on reporting obesity or overweight prevalence. Seven of the 23 articles were excluded for the following reasons: one article did not report prevalence values for obesity or overweight, 1 article reported a combined prevalence value for Ghana and Uganda, 1 article included participants older than 19 years, 3 articles reported combined prevalence values for obesity and overweight, and 2 articles were from the same sample so one was excluded. Total number of articles included in the review was 16. The PRISMA flowchart detailing the steps in obtaining the articles included in this review is shown in Figure 1.

Table 1 provides a summary of the details of the studies included in the review. All studies were published between 2012 and 2019, with 10 of them published within the last 5 years. For 9 of the studies included in the review, data collection was conducted between 2007 and 2016; 7 of the studies did not state the period during which data were collected for the studies. The studies were conducted in the various regions of Ghana as follows: 4 studies were conducted in Ashanti Region; 4 studies in Greater Accra Region; 2 studies in the Volta Region; 1 study each in the Eastern, Central, and Northern Regions; 1 study in both Ashanti and Greater Accra Regions; and 2 studies across the country. The sample sizes of the included studies ranged from 270 to 7,550 with a combined total sample size of 29,160.

### 3.1. Prevalence of Childhood Obesity in Ghana

**Figure 2:**
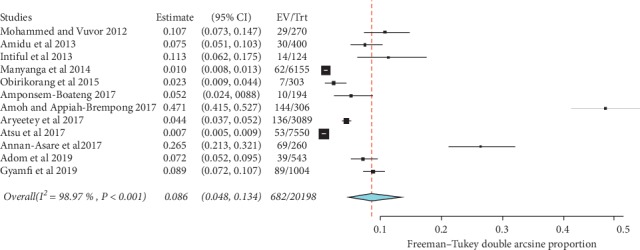
Forest plot of studies reporting the prevalence of childhood obesity in Ghana.

**Figure 3:**
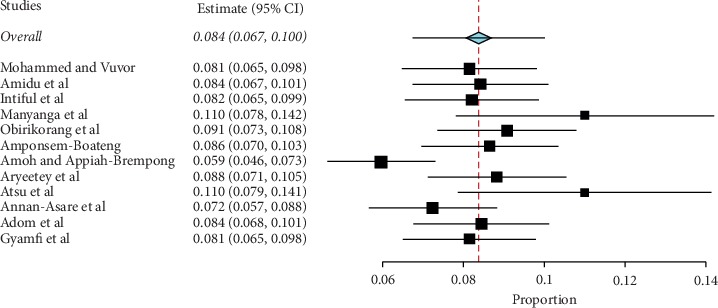
Leave-one-out sensitivity plot of studies reporting the prevalence of childhood obesity in Ghana.

**Figure 4:**
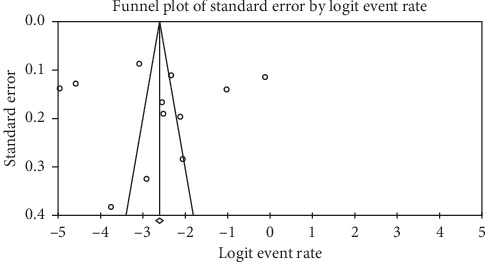
Funnel plot of studies reporting the prevalence of childhood obesity in Ghana.

Prevalence values of childhood obesity in Ghana were retrieved from 12 articles. The reported prevalence of childhood obesity in these studies ranged from 0.7% to 47.06%. The combined sample size of the 12 studies was 20,198. The pooled estimate of the prevalence of childhood obesity in Ghana was 8.6% (95% CI: 4.8%–13.4%). Heterogeneity between studies determined by *I*^2^ was 98.97% (*p* < 0.001) (Figure 2). A leave-one-out sensitivity analysis also revealed that the pooled estimate was most impacted by the prevalence values from Amoh and Appiah-Brempong [25] (Figure 3). The funnel plot revealed no publication bias in obesity prevalence reports (Figure 4). This was confirmed by Egger's test (0.4711).

Definition of obesity and overweight:

1. WHO: obesity—BMI>2 standard deviation above the WHO growth standard median (age 5–19 years); overweight—BMI > 1 standard deviation above the WHO growth standard median [49]
2. US CDC: obesity—BMI ≥ 95 percentile; overweight—BMI ≥ 85 percentile [50]
3. IOTF: obesity—BMI ≥ 30; overweight—BMI ≥ 25 [51]

^*∗*^Study locations were defined based on the previous 10 administrative regions of Ghana. Greater Accra Region is the national capital of Ghana and is in the southern part of Ghana. The Greater Accra Region is the most urbanized region in Ghana with about 90% of the populace living in urban areas but it is the smallest region by land size [52]. The Ashanti Region is in southern Ghana and is the most populous region. It is the third largest region by land size. The Ashanti Region is a largely urban region, and the economy is largely driven by extraction and processing of industrial mineral and agricultural commodities [53]. The Volta Region is in southern Ghana. It is a mostly rural region with about 64% of the populace living in rural areas. The economy of the Volta Region is largely driven by agriculture [54]. The Eastern Region is also in southern Ghana and has a predominantly rural population, with about 57% of the populace living in rural areas. The economy of the Eastern Region is hugely dominated by its high electricity generation capacity. The Northern Region is in the northern part of Ghana and is the largest region by land size. The Northern Region has a predominantly rural population, with about 70% of the populace living in rural areas. The economy of the Northern Region is driven predominantly by agriculture [55].

Prevalence values of childhood obesity among males were pooled from 6 articles included in the review. The reported prevalence of childhood obesity among males in these studies ranged from 0.6% to 9.3%. The pooled estimate of childhood obesity among males was 3.5% (95% CI: 1.4%–6.3%; *I*^2^ = 95.86%, *p* < 0.001). For females, the prevalence value for childhood obesity was pooled from 6 studies. The prevalence of childhood obesity among females reported in the studies ranged from 1.5% to 14.7%. The pooled estimate of childhood obesity among females was 4.6% (95% CI: 1.9%–8.4%; *I*^2^ = 97.08, *p* < 0.001). There was no statistically significant difference in the prevalence of obesity among males and females (*p*=0.68).

The prevalence of childhood obesity in rural locations was pooled from 3 articles. The reported prevalence of childhood obesity in rural locations ranged from 5.3% to 47.06%. The pooled estimate of childhood obesity in rural locations was 17.4% (95% CI: 1.5%–44.8%; *I*^2^ = 99.06%, *p* < 0.001). The prevalence of childhood obesity in urban locations was pooled from seven articles and ranged from 4.4% to 26.5%. The pooled estimate of childhood obesity in urban location was 8.9% (95% CI: 4.8%–14.1%; *I*^2^ = 95.44%, *p* < 0.001). There was a statistically significant difference in the prevalence of childhood obesity in rural and urban settings (*p*=0.0255).

The prevalence of childhood obesity in the Ashanti Region was pooled from 4 articles and was estimated to be 12.5% (95% CI: 1.4%–31.9%; *I*^2^ = 98.87%, *p* < 0.001). The prevalence of childhood obesity in the Greater Accra Region was also pooled from 4 articles and was estimated to be 12.1% (95% CI: 7.9%–17.0%; *I*^2^ = 74.09 %, *p* < 0.001).

### 3.2. Prevalence of Childhood Overweight in Ghana

**Figure 5:**
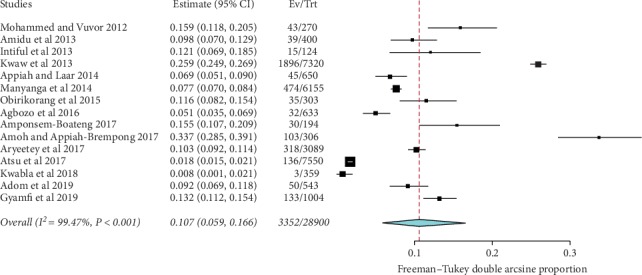
Forest plot of studies reporting the prevalence of childhood overweight in Ghana.

**Figure 6:**
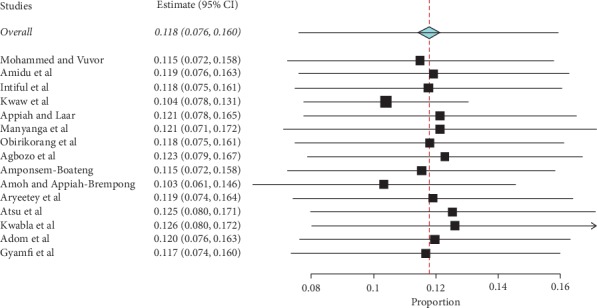
Leave-one-out plot sensitivity plot of studies reporting the prevalence of childhood overweight in Ghana.

**Figure 7:**
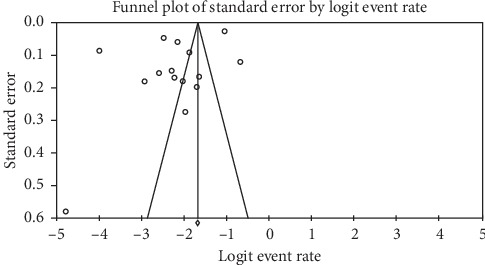
Funnel plot of studies reporting the prevalence of childhood overweight in Ghana.

Prevalence values of childhood overweight in Ghana were retrieved from 15 articles. The total sample size was 28,900. The reported prevalence of childhood overweight in these studies ranged from 0.8% to 33.7%. The pooled estimate of the prevalence of childhood overweight in Ghana was 10.7% (95% CI: 5.9–16.6). Heterogeneity between studies determined by *I*^2^ was 99.47% (*p* < 0.001) (Figure 5). A leave-one-out sensitivity analysis also revealed that the pooled estimate was most impacted by the prevalence values from Amoh and Appiah-Brempong [25] (Figure 6). The funnel plot revealed publication bias, depicted by the asymmetrical display of prevalence values reported by the various studies (Figure 7); this was confirmed by Egger's test (*p*=0.043).

Prevalence values of childhood overweight among males were pooled from 6 articles. The reported prevalence of childhood overweight among males in these studies ranged from 2.1% to 16.3%. The pooled estimate of childhood overweight among males was 7.2% (95% CI: 4.0%–11.4%; *I*^2^ = 96.48%, *p* < 0.001). For females, the prevalence value for childhood overweight was pooled from 6 studies. The prevalence of childhood overweight among females reported in the studies ranged from 1.4% to 19.4%. The pooled estimate of childhood overweight among females was 10.9% (95% CI: 4.0%–20.4%; *I*^2^ = 99.04%, *p* < 0.001). There was no statistically significant difference in the prevalence of overweight among males and females (*p*=0.181).

The prevalence of childhood overweight in rural locations was pooled from 5 articles and ranged from 0.8% to 33.7%. The pooled estimate of childhood overweight in rural locations was 11.8% (95% CI: 4.1%–22.7%; *I*^2^ = 98.01 %, *p* < 0.001). The prevalence of childhood overweight in urban locations was estimated from eight articles and ranged from 5.1% to 25.9%. The pooled estimate of childhood overweight in urban location was 12.0% (95% CI: 6.3%–19.1%; *I*^2^ = 98.84 %, *p* < 0.001). There was no significant difference in the prevalence of overweight in rural and urban settings (*p*=0.692).

The prevalence of childhood overweight in the Ashanti Region was pooled from 4 articles and was estimated to be 17.7% (95% CI: 9.7%–27.6%; *I*^2^ = 95.33%, *p* < 0.001). The prevalence of childhood overweight in the Greater Accra Region was also pooled from 3 articles and was estimated to 12.1% (95% CI: 7.7%–17.0%; *I*^2^ = 74.09 %, *p*=0.021).

## 4. Discussion

The current study provides the first systematic review and meta-analysis on childhood obesity and overweight in Ghana. The overall prevalence of childhood obesity and overweight in Ghana was 8.6% and 10.7%, respectively. This is comparable to the prevalence values reported by other regional studies in Africa [56]. These prevalence values are however higher compared to the expected value for developing countries and the world at large. Combined prevalence of obesity and overweight in developing countries was 6.1% in 2010 and is expected to reach 8.6% by 2020 [9]. This highlights the extent of the childhood obesity and overweight burden in Ghana and the need for public health interventions geared towards fighting this public health issue.

Over the past decades, the focus of almost all nutritional programs in developing countries has been on undernutrition, as undernutrition is well-established and recognized problem of the developing world. While undernutrition still remains a problem for some developing countries, another nutritional problem being faced by most developing countries is obesity and overweight. For a long time, obesity has been seen as a problem of affluent and developed countries, but this is changing. Due to urbanization and economic development, the prevalence of childhood obesity and obesity in general is increasing in developing countries like Ghana. The rise in obesity in developing countries has largely been influenced by changes in dietary patterns. People are no longer eating the traditional nutritious food as consumption of easy and readily available high-fat and caloric content foods is fast increasing. In Ghana, there has been a rise in the fast food franchise especially in big cities, with a lot of Ghanaians patronizing fast food restaurants. The influence of fast food consumption on rising cases of obesity is well established in both developed and developing countries [57–62]. Another factor fueling the rise in obesity is the increase in sedentary lifestyle of people, especially in urban centers. There has been a decrease in physical activities in both developed and developing countries [13]. Advancement in technology has led to relative ease of doing things and moving around; therefore, there is a considerable decrease in opportunities for physical activities [14, 15]. Children spend more time indoors than they do outdoors, playing video games, and partaking in other indoor activities [16–18]. The energy imbalance resulting from the increased high caloric food intake and decreased physical activities is the driving force behind the increase in obesity and overweight over the past decades [19]. The paradoxical coexistence of undernutrition and overnutrition in most developing countries is disturbing public health problem, as most groups within these developing countries have low socioeconomic strength and hence will lack the financial ability to afford nutritionally rich food but are at the same time more likely to consume cheaper energy-dense foods that are known to cause obesity [63].

Obesity in general seems to be a rising problem for Ghana. A recent review reported the prevalence of obesity and overweight in adults 18 years above to be 17.1% and 25.6%, respectively. Thus, Ghana appears to have a high prevalence of both childhood and adulthood obesity and overweight, and these patterns will continue to increase given the current trend of dietary changes in Ghana. The high prevalence of childhood and adulthood obesity is a concern to clinical and public health experts, nutritional scientists, and government due to the potential effects it could have on the health of citizens. With the high prevalence of obesity, Ghana stands the unfortunate possibility of rising numbers of diabetes, hypertension, and other cardiovascular diseases which are already major reasons for frequent visits to health facilities among the older population.

Contrary to other studies, although point estimates for childhood obesity and overweight were generally higher in females than in males, no significant gender difference in childhood obesity or overweight was found in the current meta-analysis. Reports on the gender difference in childhood obesity or overweight prevalence are inconsistent; some studies report higher prevalence in males [64, 65], others report higher prevalence in females [66]while some report no gender difference in childhood obesity or overweight prevalence [67]. In a similar study involving a meta-analysis of obesity and overweight among school-going children in Africa, no gender difference in obesity or overweight prevalence was reported [56]. While there is no coherent gender-related pattern in childhood obesity and overweight, a consistent gender-related pattern is observed in obesity and overweight among adults, where the prevalence is frequently higher in females compared to males [1, 67–69]. This suggests an age-gender interaction in obesity and overweight [64, 70].

In contrast to other studies, the current review reports a significantly higher prevalence of obesity in rural areas than in urban areas. Almost all other studies consistently report higher estimates of obesity prevalence in urban areas compared to rural areas [71–73]. The apparently contrary report from the current review might be to the low number of studies used in pooling the prevalence estimates of obesity for the rural area. As such, one of the studies, which reported a higher prevalence value, skewed the pooled estimate.

A key limitation of this review is the regional imbalance in the studies included. Even though efforts were made to include studies from across the country, over half of the studies used in this review were conducted in two regions (Ashanti and Greater Accra Regions). Also, some of the studies included were not originally designed to assess obesity and overweight prevalence and as such might not have put in place measures to prevent bias in reporting the prevalence. Also, it is acknowledged that some relevant studies might have been missed as they might have been published in local journals that did not appear in our literature search. Most of the studies did not report prevalence values for obesity or overweight across sociodemographic characteristics such as gender and setting. Hence, not enough articles were used in calculating the pooled estimates across those sociodemographic characteristics. Despite all these limitations, the prevalence values for childhood obesity and overweight estimated by this review should be a close representation of the situation within the current or immediate past decade as sampling and publication of all studies occurred within the current or immediate past decade.

## 5. Conclusion

The high prevalence of childhood obesity and overweight estimated in this review is of worrying concern. It is a significant public health problem that has implications on the health of present and future generations in Ghana and as such calls for proactive measures to be put in place. Also, the driving forces behind the increasing prevalence of childhood obesity in Ghana need to be investigated.

## Figures and Tables

**Table 1:** Descriptive characteristics of studies included in the review.

Study no.	Authors	Publication year	Data collection years	Location^∗^	Setting	Study design	Sample size	Age range of participants (years)	Definition criteria	Prevalence (%)	Quality score
1	Mohammed and Vuvor [37]	2012	Not stated	Greater Accra Region	Urban	Cross-sectional	270	5–15	WHO	Overweight: 15.8 Obesity: 10.9	6
2	Amidu et al. [38]	2013	2012/2013	Northern Region	Urban	Cross-sectional	400	6–12	US CDC	Overweight: 9.8 Obesity: 7.5	8
3	Intiful et al. [39]	2013	Not stated	Greater Accra Region	Urban	Cross-sectional	124	8–10	WHO	Overweight: 12.1 Obesity: 11.3	4
4	Kwaw et al. [40]	2013	2011/2012	Central Region	Urban	Cross-sectional	7320	11–14	WHO	Overweight: 25.9	5
5	Appiah and Laar [41]	2014	2014	Volta Region	Rural	Cross-sectional	650	10–19	WHO	Overweight: 6.9	9
6	Manyanga et al. [42]	2014	2007	Across the country	General population	Cross-sectional	6155	Not stated	WHO	Overweight: 7.7 Obesity: 1.0	8
7	Obirikorang et al. [43]	2015	2013/2014	Ashanti Region	Urban	Cross-sectional	303	6–12	US CDC	Overweight: 11.6 Obesity: 2.3	8
8	Agbozo et al. [44]	2016	2014	Volta Region	Urban	Cross-sectional	633	3–12	WHO	Overweight: 5.06	9
9	Amponsem-Boateng [45]	2017	Not stated	Ashanti Region	Rural	Cross-sectional	194	1–8	WHO	Overweight: 15.5 Obesity: 5.3	2
10	Amoh and Appiah-Brempong [25]	2017	2014/2015	Ashanti Region	Rural	Cross-sectional	306	14–19	IOTF	Overweight: 33.66 Obesity: 47.06	6
11	Aryeetey et al. [46]	2017	2009/2012	Greater Accra and Ashanti Regions	Urban	Cross-sectional	3089	9–15	WHO	Overweight: 10.3 Obesity: 4.4	9
12	Atsu et al. [23]	2017	Not stated	Across the country	General population	Cross-sectional	7550	0–5	WHO	Overweight: 1.8 Obesity: 0.7	9
13	Annan-Asare et al. [26]	2017	Not stated	Greater Accra Region	Urban	Cross-sectional	260	11–15	WHO	Obesity: 26.5	8
14	Kwabla et al. [24]	2018	2016	Eastern Region	Rural	Cross-sectional	359	5–12	WHO	Overweight: 0.8	9
15	Adom et al. [47]	2019	Not stated	Greater Accra Region	Urban	Cross-sectional	543	8–11	WHO	Overweight: 9.2 Obesity: 7.2	9
16	Gyamfi et al. [48]	2019	Not stated	Ashanti Region	Rural	Cross-sectional	1004	5–17	WHO	Overweight: 13.2 Obesity: 8.85	8

WHO–World Health Organization; US CDC: United States Centers for Disease Control and Prevention; IOTF: International Obesity Task Force.

## Supplementary Materials

Supplementary Materials Forest plot subanalysis of gender and study setting.
