# Supplementary material for: Childhood Obesity and Overweight in Ghana: A Systematic Review and Meta-Analysis
Source: J Nutr Metab. 2020 Apr 8;2020:1907416. doi: 10.1155/2020/1907416 (PMC7168721; doi:10.1155/2020/1907416)
Supplement: Supplementary Materials — Forest plot subanalysis of gender and study setting. [file 1907416.f1.pdf]

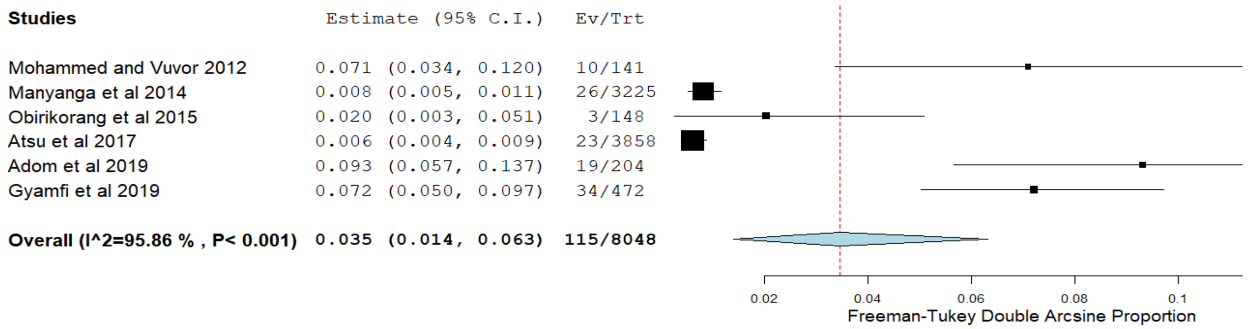

Figure 1.0 Forest plot of studies reporting prevalence of childhood obesity among males in Ghana

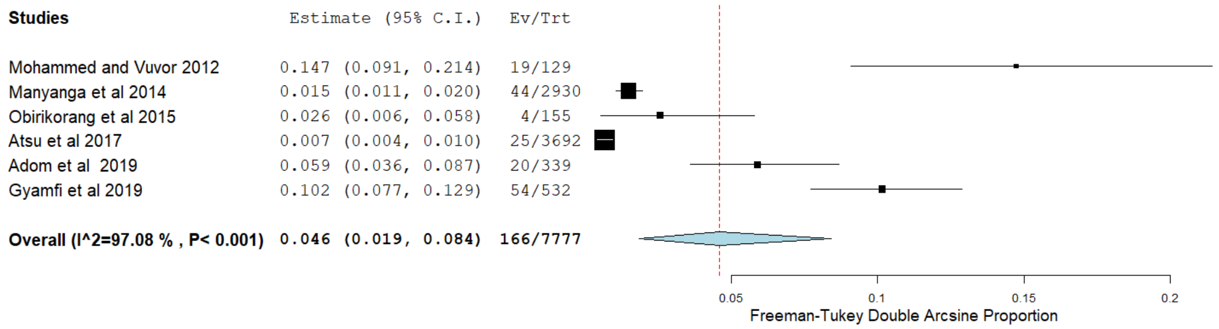

Figure 2.0 Forest plot of studies reporting prevalence of childhood obesity among females in Ghana

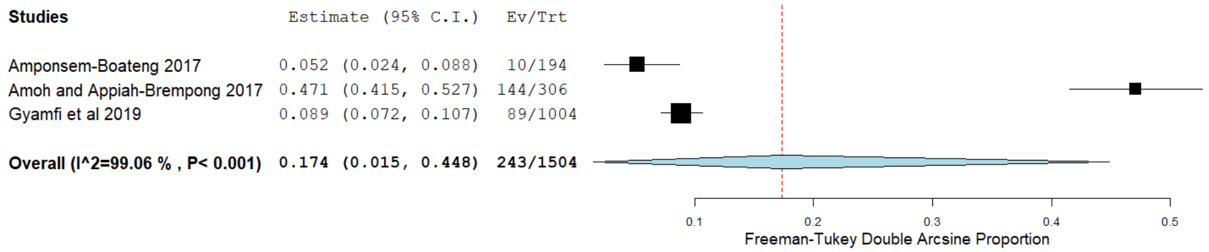

Figure 3.0 Forest plot of studies reporting prevalence of childhood obesity in rural settings in Ghana

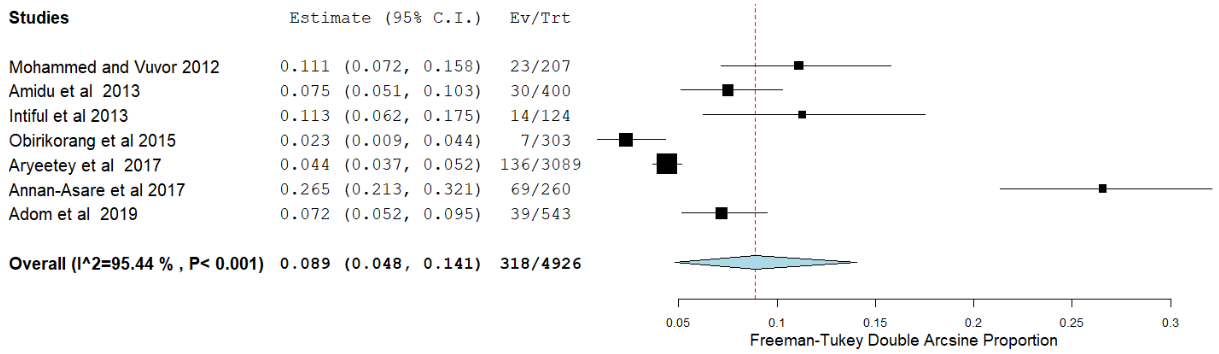

Figure 4.0 Forest plot of studies reporting prevalence of childhood obesity in urban settings in Ghana

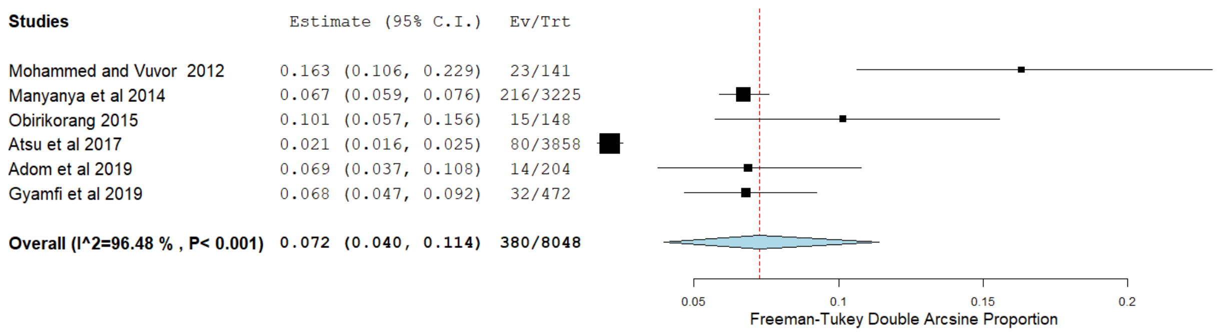

Figure 5.0 Forest plot of studies reporting prevalence of childhood overweight among males in Ghana

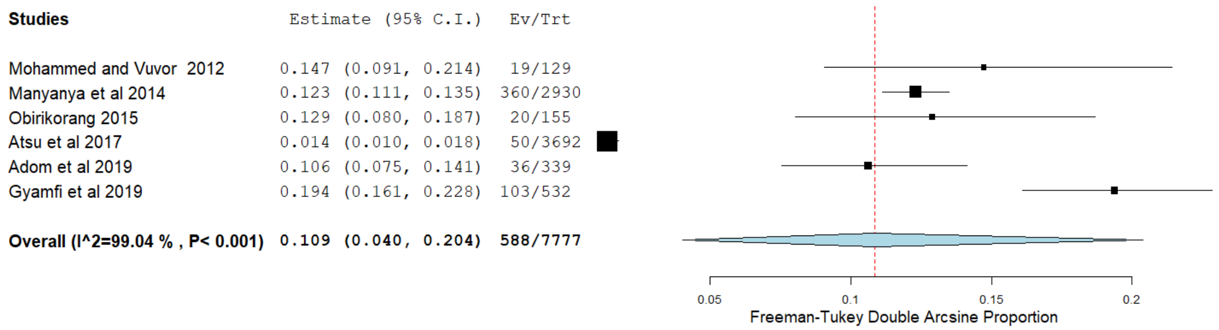

Figure 6.0 Forest plot of studies reporting prevalence of childhood overweight among females in Ghana

Studies	Estimate (95% C.I.)	Ev/Trt
Appiah and Laar 2014	0.069 (0.051, 0.090)	45/650
Amponsem-Boateng 2017	0.155 (0.107, 0.209)	30/194
Amoh and Appiah-Brempong 2017	0.337 (0.285, 0.391)	103/306
Kwabla 2018	0.008 (0.001, 0.021)	3/359
Gyamfi et al 2019	0.132 (0.112, 0.154)	133/1004
**Overall (I^2=98.01 % , P< 0.001)**	**0.118 (0.041, 0.227)**	**314/2513**

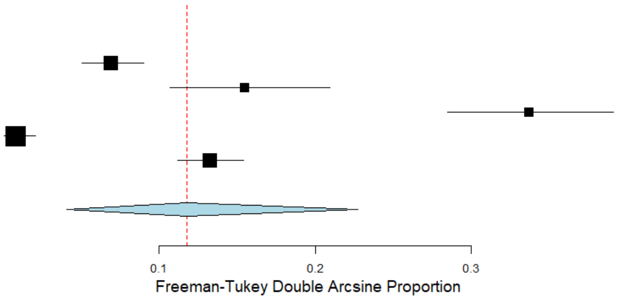

Figure 7.0 Forest plot of studies reporting prevalence of childhood overweight in rural settings in Ghana

Studies	Estimate (95% C.I.)	Ev/Trt
Mohammed and Vuvor 2012	0.159 (0.118, 0.205)	43/270
Amidu et al 2013	0.098 (0.070, 0.129)	39/400
Kwaw et al 2013	0.259 (0.249, 0.269)	1896/7320
Intiful et al 2013	0.121 (0.069, 0.185)	15/124
Obirikorang et al 2015	0.116 (0.082, 0.154)	35/303
`Agbozo et al 2016	0.051 (0.035, 0.069)	32/633
Aryeetey et al 2017	0.103 (0.092, 0.114)	318/3089
Adom et al 2019	0.092 (0.069, 0.118)	50/543
**Overall (I^2=98.84 % , P< 0.001)**	**0.120 (0.063, 0.191)**	**2428/12682**

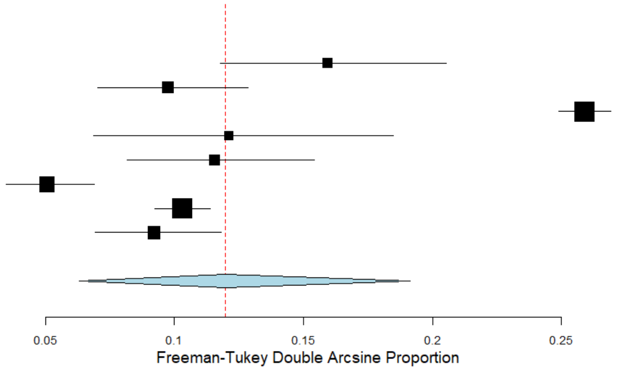

Figure 8.0 Forest plot of studies reporting prevalence of childhood overweight in urban settings Ghana
